# Nuclear Localization of HopA1_Pss61_ Is Required for Effector-Triggered Immunity

**DOI:** 10.3390/plants10050888

**Published:** 2021-04-28

**Authors:** Hobin Kang, Quang-Minh Nguyen, Arya Bagus Boedi Iswanto, Jong Chan Hong, Saikat Bhattacharjee, Walter Gassmann, Sang Hee Kim

**Affiliations:** 1 Division of Applied Life Science (BK21 Four Program), Plant Molecular Biology and Biotechnology Research Center, Gyeongsang National University, 501 Jinju-daero, Jinju 52828, Korea; hegood123@naver.com (H.K.); nguyenquangminh4595@gmail.com (Q.-M.N.); aryabagus62@gmail.com (A.B.B.I.); jchong@gnu.ac.kr (J.C.H.); 2 Division of Life Science, Gyeongsang National University, 501 Jinju-daero, Jinju 52828, Korea; 3 Laboratory of Signal Transduction and Plant Resistance, UNESCO—Regional Centre for Biotechnology (RCB), NCR—Biotech Science Cluster, 3rd Milestone, Faridabad-Gurgaon Expressway, Faridabad 121 001, India; saikat@rcb.res.in; 4 Division of Plant Sciences, Christopher S. Bond Life Sciences Center and Interdisciplinary Plant Group, University of Missouri, Columbia, MO 65211, USA; gassmannw@missouri.edu

**Keywords:** effector-triggered immunity, *HopA1_Pss61_*, RPS6, resistant protein, effector

## Abstract

Plant resistance proteins recognize cognate pathogen avirulence proteins (also named effectors) to implement the innate immune responses called effector-triggered immunity. Previously, we reported that *hopA1* from *Pseudomonas syringae* pv. *syringae* strain 61 was identified as an *avr* gene for *Arabidopsis thaliana*. Using a forward genetic screen approach, we cloned a *hopA1-*specific TIR-NBS-LRR class disease resistance gene, *RESISTANCE TO PSEUDOMONAS SYRINGAE6* (*RPS6*). Many resistance proteins indirectly recognize effectors, and RPS6 is thought to interact with *HopA1_Pss61_* indirectly by surveillance of an effector target. However, the involved target protein is currently unknown. Here, we show RPS6 is the only R protein that recognizes *HopA1_Pss61_* in Arabidopsis wild-type Col-0 accession. Both RPS6 and *HopA1_Pss61_* are co-localized to the nucleus and cytoplasm. *HopA1_Pss61_* is also distributed in plasma membrane and plasmodesmata. Interestingly, nuclear localization of *HopA1_Pss61_* is required to induce cell death as NES-*HopA1_Pss61_* suppresses the level of cell death in *Nicotiana benthamiana*. In addition, in planta expression of *hopA1_Pss61_* led to defense responses, such as a dwarf morphology, a cell death response, inhibition of bacterial growth, and increased accumulation of defense marker proteins in transgenic Arabidopsis. Functional characterization of *HopA1_Pss61_* and RPS6 will provide an important piece of the ETI puzzle.

## 1. Introduction

Plants are challenged by a wide variety of pathogens. However, they have evolved sophisticated immune systems to protect themselves from pathogen infections. [1,2]. The plant immune systems involve two different actions. One is pattern-triggered immunity (PTI), and the other is effector-triggered immunity (ETI) [3,4]. PTI is typically activated by the recognition of pathogen-associated molecular patterns (PAMPs) with pattern recognition receptors (PRRs) in the plants. This recognition process is required to promote defense signaling pathways, such as the activation of mitogen-activated protein kinase (MAPK) cascades, reactive oxygen species, ion channel opening, callose deposition, and defense-related genes [4,5,6]. However, to encounter PTI, pathogens deploy several infectious effectors. Subsequently, plants utilize a second layer of defense through the activation of resistance (R) proteins; this mode is known as ETI response [3,7].

At the ETI mode, pathogen effectors are recognized by resistance (R) proteins, which are notably found in the form of nucleotide-binding (NB) domain and leucine-rich repeat (LRR) containing receptors (NLR) [8,9]. The NB domain of R proteins has sequence homology to NB domains of apoptosis regulators such as apoptotic protease activating factor-1 (Apaf-1) and cell death protein 4 (Ced4), suggesting the NB domain is involved in ATP binding and hydrolysis. For instance, recent studies of ZAR1 structure indicate that the dATP/ATP incorporation to the NB domain induces the ZAR1 pentameric oligomerization during immune response [10]. The LRR domain is a common motif of 20–30 amino acids in length, represented in over 2000 proteins from viruses to eukaryotes [7,11,12]. The NB-LRR class of *R* genes can be grouped into two families, coiled-coil (CC)-NB-LRR (CNL) and Toll-interleukin-1 receptor (TIR)-NB-LRR (TNL). The plant TIR domain has structural and functional similarity to the cytoplasmic domain of the Drosophila Toll and mammalian interleukin-1 transmembrane receptor. Similar functionality and organization of plant TIR-type R proteins and animal Toll-like receptors (TLRs) is thought to represent convergent evolution of the innate immunity pathway of these kingdoms [9]. Recent studies demonstrate the structural similarity between crystals of human SARM1^TIR^ and plant NLR-TIRs. Like SARM1^TIR^, plant L6^TIR^, RBA1^TIR^, RPS4^TIR^, and other plant TIRs also act as NADases due to a conserved putative catalytic glutamic acid. Moreover, the NAD+ cleavage activity of plant TIRs is TIR-oligomerization dependent and important for NLR-mediated immunity [13,14].

One of the intriguing issues in the study of plant innate immunity is the mode of recognition between host R proteins and pathogen effectors to elicit defense cascades. R protein can interact with cognate effector protein directly as implied by the gene-for-gene hypothesis. For instance, L5 and L6 flax TNL R proteins bind to the AvrL567 effectors from flax rust fungus [15,16]. Another TNL type R protein, RPP1, is also known to directly recognize ATR1 derived from oomycete pathogen *Hyaloperonospora arabidopsidis* [17]. In those cases, the LRR domain plays a role in effector recognition. The direct recognition system can be used to select pathogen effectors [18]. Another hypothesis has arisen where a plant R protein indirectly recognizes a corresponding effector protein by monitoring effector perturbation of a host target (guardee or decoy) [3,7]. In this system, R proteins are considered to survey proteins which effectors target. For example, RPS2 and RPM1 recognize diverse effectors, AvrRpt2, AvrB, and AvrRpm1, by monitoring modification of RPM1-interacting protein 4 (RIN4) [19,20]. RPS5 is typically activated by the proteolytic cleavage of a decoy protein, PBS1, by the pathogen-secreted protease AvrPphB [21,22]. Moreover, the recognition specificity of RPS5 can be expanded by substituting the cleavage sequence of AvrPphB within PBS1 to those of other pathogen proteases, suggesting that a plant decoy protein can be engineered to broaden the specificity of R protein [23]. The guard model has provided a mechanistic understanding of the recognition specificity mechanism for the CNL proteins RPM1, RPS2, and RPS5. 

RPS6, a TNL type R protein, recognizes the presence of avirulence effector HopA1 from *Pseudomonas syringae* pv. *syringae* strain 61 (after this referred to as *HopA1_Pss61_*) but not HopA1 from *P. syringae* pv. *tomato* strain DC3000 (after this referred to as HopA1_DC3000_) [24,25,26]. Although a recent study implies that RPS6 indirectly recognizes the presence of *HopA1_Pss61_* in the plant [27], the identity of the effector target protein, possibly guardee or decoy, is still unknown. 

Here, we characterized plant immune responses triggered by *HopA1_Pss61_*. Using genetic T-DNA mutant isolation and bacterial pathogenesis assay within a cluster of *R* genes and contradictory gene models, we identified RPS6 functions as the R protein for the recognition of *HopA1_Pss61_* in Col-0. Moreover, we showed that a nuclear pool of *HopA1_Pss61_* is necessary to induce cell death and defense responses. Indeed, defense responses, such as a dwarf morphology, a cell death response, bacterial growth inhibition, and increased accumulation of defense marker proteins, were boosted in the transgenic Arabidopsis plants expressing *hopA1_Pss61_*.

## 2. Results

### 2.1. RPS6 (At5g46470) Recognizes *HopA1_Pss61_* in Arabidopsis Col-0 Accession

**Figure 1:**
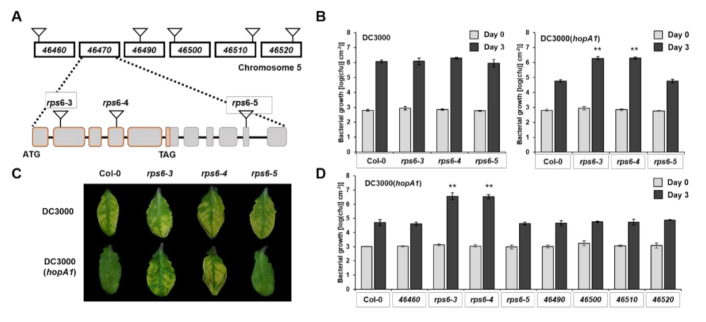
*RPS6* (*At5g46470*) recognizes *HopA1_Pss61_* in Arabidopsis Col-0 accession. (**A**) Schematic diagram of chromosome 5 around *RPS6* (*At5g46470*) and position of T-DNA insertion along the genes. *rps6-3* and *rps6-4* possess T-DNA insertion in exon 2 and exon 4, respectively, while *rps6-5* in exon 10; (**B**) In planta bacterial growth was measured in Col-0, *rps6-3*, *rps6-4*, and *rps6-5* on day 0 (gray columns) and day 3 (black columns) after inoculation with DC3000 (left) and DC3000(*hopA1_Pss61_*) (right); (**C**) Plants were inoculated with a bacterial suspension at a density of 5 × 10^6^ cfu/mL suspensions of DC3000 (top) and DC3000(*hopA1_Pss61_*) (bottom). Photos were taken 4 days post-inoculation; (**D**) In planta bacterial growth was measured in Col-0, *At5g46460, At5g46490, At5g46500, At5g46510, At5g46520, rps6-3*, *rps6-4*, and *rps6-5* on day 0 (gray columns) and day 3 (black columns) after inoculation with DC3000(*hopA1_Pss61_*); (**B**,**D**) Plants were inoculated with a bacterial suspension at a density of 2 × 10^5^ cfu/mL. Values represent averages of cfu/cm^2^ leaf tissue from quadruplicate samples, and error bars denote standard deviation. Asterisks indicate that the growth of DC3000(*hopA1_Pss61_*) on day 3 was significantly different between Col-0 and *rps6-3* or *rps6-4*, as determined by a two-tailed Student’s *t*-test (** *p* < 0.01). This experiment was repeated twice with similar results.

HopA1 is found in both avirulent *P. syringae* pv. *syringae* strain 61 (denoted *HopA1_Pss61_*) and virulent *P. syringae* pv. *tomato* strain DC3000 (denoted HopA1_DC3000_). Previous studies have suggested that RPS6 in Arabidopsis RLD accession recognizes *HopA1_Pss61_* but not HopA1_DC3000_ [26]. Here, we expanded our understanding of HopA1-triggered RPS6-mediated immune responses in wild-type Col-0 accession. *RPS6* (*At5g46470*) is positioned at the bottom of chromosome 5, in which six TNL class resistance genes are clustered around *RPS6* (Figure 1A). In our former study, a pSHK103 construct used in the RLD background *rps6-1* complementation assay possessed *At5g46460* as well as *At5g46470* [26]. In addition, Takagi and colleagues found a polycistronic transcript of *At5g46460* and *At5g46470* [27]. To eliminate the possibility that *At5g46460* recognizes *HopA1_Pss61_* and to clarify that *RPS6* (*At5g46470*) is the only *R* gene that senses the *HopA1_Pss61_*, we isolated homozygous T-DNA insertion mutants of *At5g46460, At5g46490, At5g46500, At5g46510, At5g46520*, and *At5g46470* (*rps6-3, rps6-4*, and *rps6-5*) in the Col-0 background in which *At5g46460* encodes a pentatricopeptide repeat protein, whereas others belong to TNL class R proteins (Figure 1A). In planta bacterial growth assays were performed to quantify the level of resistance. The T-DNA insertion lines of *At5g46470, rps6-3 and rps6-4,* had approximately 50-fold higher DC3000(*hopA1_Pss61_*) growth than Col-0, but they were as susceptible as Col-0 in response to virulent DC3000 (Figure 1B,C). However, the T-DNA insertion lines of *RPS6* surrounding genes, including *At5g46460*, *At5g46490*, *At5g46500*, *At5g46510*, and *At5g46520*, were as resistant as Col-0 against DC3000(*hopA1_Pss61_*) (Figure 1D). These results suggest that *RPS6* (*At5g46470*) solely recognizes *HopA1_Pss61_* and that other *TNLs* and *At5g46460* are not required for the *HopA1_Pss61_*-recognition.

*RPS6* possesses a long 3′ untranslated region (UTR) containing six exons and five introns with a length of approximately 3 kb. The long 3′ UTR is a typical characteristic of nonsense-mediated mRNA decay (NMD) target, and *RPS6* is required for autoimmunity in NMD-deficient mutant *smg7* [28,29]. As shown in Figure 1B, *rps6-5* with a T-DNA in the tenth exon of *RPS6* (at position 1789 of its 3′UTR) behaves like Col-0 in response to DC3000(*hopA1_Pss61_*). Consistent with these, *RPS6* transcripts were detected in Col-0 and *rps6-5* but not in *rps6-3* and *rps6-4* (Supplementary Figure S1). This result demonstrates a 1789 bp 3′ UTR of RPS6 is sufficient to confer resistance to DC3000(*hopA1_Pss61_*).

### 2.2. HopA1_Pss61_ Targets to the Nucleus, Cytoplasm, Plasma Membrane, and Plasmodesmata

**Figure 2:**
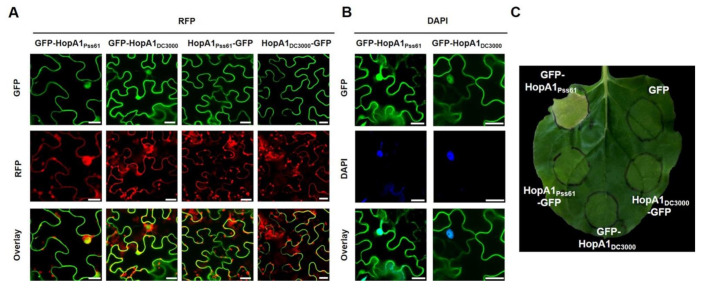
GFP-*HopA1_Pss61_*, but not *HopA1_Pss61_*-GFP, targets to the nucleus and induces cell death. (**A**) *GFP-hopA1_Pss61_*, *hopA1_Pss61-_GFP*, *GFP-hopA1_DC3000_*, and *hopA1_DC3000-_GFP* fusion constructs (from left to right) were transiently expressed in *N. benthamiana* leaves. RFP was co-expressed to label the nucleus and cytoplasm of transiently transformed cells. Cells expressing fusion proteins were analyzed 2 days after infiltration by an Olympus fluoview FV1000 confocal microscope under GFP fluorescence (top), RFP fluorescence (middle), and GFP/RFP overlay (bottom). Scale bar: 20 μm; (**B**) Nuclear localization of GFP-*HopA1_Pss61_* and GFP-HopA1_DC3000_ were confirmed by 4′,6-diamidino-2-phenylindole (DAPI) staining. Two days after infiltration, cells expressing proteins were analyzed by an Olympus fluoview FV1000 confocal microscope under GFP fluorescence (top), DAPI (middle), and GFP/DAPI overlay (bottom). Scale bar: 20 μm. (**C**) Only GFP-*HopA1_Pss61_* induced cell death in *N. benthamiana*. GFP-*HopA1_Pss61_*, *HopA1_Pss61_*-GFP, GFP-hopA1_DC3000_, and hopA1_DC3000_-GFP fusion constructs were transiently expressed in *N. benthamiana* leaves using Agrobacterium adjusted to an OD_600_ of 0.2. Phenotypes were recorded at 4 days post-inoculation.

The amino acid sequences of *HopA1_Pss61_* and HopA1_DC3000_ are 57% identical, and diverged amino acids are distributed throughout the proteins [26]. In order to investigate the subcellular localization of the *HopA1_Pss61_* and HopA1_DC3000_ effector proteins, we fused a GFP tag to the N- and C- terminus of each HopA1 driven by the 35S promoter. The HopA1 derivatives, GFP-*HopA1_Pss61_*, *HopA1_Pss61_*-GFP, GFP-HopA1_DC3000_, and HopA1_DC3000_-GFP, were transiently expressed in *N. benthamiana* leaves using agroinfiltration. GFP-*HopA1_Pss61_*, GFP at the N-terminus of *HopA1_Pss61_*, was localized to the nucleus, cytoplasm, and plasma membrane (PM) (Figure 2A,B, Supplementary Figures S2 and S3). In the nucleus, GFP-*HopA1_Pss61_* was detected not only in the nucleoplasm but also in the nucleolus (Figure 2A). A similar localization pattern was observed in GFP-HopA1_DC3000_ (Figure 2A). Surprisingly, HopA1_Pss61_-GFP and HopA1_DC3000_-GFP were found in the PM but not in the nucleus (Figure 2A) and co-localized with PM localized protein PBS1-mCherry (Supplementary Figure S2) [30], suggesting C-terminally fused GFP inhibits the nuclear localization of both HopA1s. The expression of HopA1 derivatives was detected by Western blot analysis, confirming that the GFP fusion proteins were full-length (Supplementary Figure S5). 

In addition, *HopA1_Pss61_*-GFP was observed in punctate spots around the cell periphery, indicating plasmodesmata (PD) localization (Supplementary Figure S3C). PD is considered as a space that can act as a passage between cells [31,32]. To carefully investigate the *HopA1_Pss61_* localization, we co-expressed GFP-*HopA1_Pss61_* with PDLP5-RFP, a marker protein of PD [33]. As shown in Supplementary Figure S3, *HopA1_Pss61_* and PDLP5 were co-localized in the PD and PM in the plasmolysis condition. In addition, the co-localization of *HopA1_Pss61_* and PBS1 to PM was observed (Supplementary Figure S2). Taken together, we conclude that *HopA1_Pss61_* is distributed not only in nucleocytoplasm but also in PM and PD.

### 2.3. Nuclear Localization of HopA1_Pss61_ Induces Cell Death Responses

The pHIR11 cosmid containing *hopA1* and *Pss61* type III secretion system genes, when expressed in *P. fluorescens*, can induce a robust hypersensitive response (HR) in tobacco [34,35]. To test whether HopA1 alone can elicit cell death, agrobacteria expressing the GFP-tagged *hopA1* constructs used in Figure 2A were infiltrated into *N. benthamiana* leaves. Interestingly, despite the lower level of protein expression compared with other derivatives in the immunoblot (Supplementary Figure S5), only GFP-*HopA1_Pss61_*, which targets the nucleus, was capable of inducing an HR-like cell death four days after infiltration, whereas *HopA1_Pss61_*-GFP, GFP-HopA1_DC3000_, and HopA1_DC3000_-GFP failed to exhibit the cell death response (Figure 2B). To confirm that GFP-*HopA1_Pss61_*-induced cell death is GFP-tagging-independent, we overexpressed *HopA1_Pss61_* with another epitope in *N. benthamiana*. Indeed, similar to GFP-*HopA1_Pss61_*, N-terminally HA-tagged HopA1_Pss61_ triggered a cell death response (Supplementary Figure S4). These results raise the possibility that effector *HopA1_Pss61_*, not HopA1_DC3000_, is recognized by tobacco and that the nuclear pool of GFP-*HopA1_Pss61_* is essential for the cell death induction.

For the confirmation of *HopA1_Pss61_* expression in the nucleus, a nuclear fractionation assay was performed. Proteins were extracted from *N. benthamiana* expressing GFP-*HopA1_Pss61_* and fractionated. Western blot showed *HopA1_Pss61_* was accumulated in both nuclear and cytoplasmic (non-nuclear) fractions (Supplementary Figure S6), consistent with the nucleocytoplasmic distribution of *HopA1_Pss61_* in the confocal microscopy analysis. Histone H3 and PEPC were detected only in the nucleus and cytoplasm, respectively, indicating a high degree of enrichment of the indicated compartment in our fractions.

**Figure 3:**
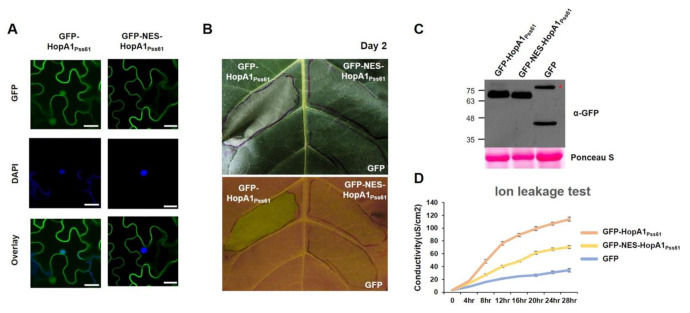
The nuclear pool of *HopA1_Pss61_* is required for ETI responses. (**A**) GFP-*HopA1_Pss61_* and GFP-NES-*HopA1_Pss61_* constructs were transiently expressed in *N. benthamiana* leaves using Agrobacterium adjusted to an OD_600_ of 0.2. Two days after infiltration, leaf discs were stained with 4′,6-diamidino-2-phenylindole (DAPI) for nucleus detection. Two days after infiltration, cells expressing fusion proteins were analyzed by an Olympus fluoview FV1000 confocal microscope under GFP fluorescence (top), DAPI (middle), and GFP/DAPI overlay (bottom). Scale bar: 20 μm. (**B**) GFP-hopA1_Pss61,_ GFP-NES-*HopA1_Pss61_*, and GFP constructs were transiently expressed in *N. tabacum* cv. *Xanthi* leaves using Agrobacterium adjusted to an OD_600_ of 0.2. The photographs were taken under visible light (top) and UV light (bottom) at 2 days post-inoculation; (**C**) Detection of HopA1 protein by Western blot. Expression of GFP-*HopA1_Pss61_*, GFP-NES-*HopA1_Pss61_*, GFP in samples shown in (**A**) and (**B**) was confirmed by Western blot with anti-GFP antibody. Total protein was extracted from six leaf discs at 2 days post-inoculation. Total protein staining (Ponceau S) confirmed equal loading in Western blot analysis. The asterisk indicates a non-specific band cross-reacting with the anti-GFP antibody; (**D**) Quantification of cell death triggered by the hopA1 constructs described in (**B**) using electrolyte leakage. Error bars indicate standard deviation. Conductivity was measured at the indicated time points. This experiment was repeated twice with a similar result.

To analyze the connection between the *HopA1_Pss61_* localization and function, we added the nuclear export signal (NES) that originated from the HIV-1 Rev protein to the N terminal of *HopA1_Pss61_*. First, we put to test the localization of *HopA1_Pss61_* in the presence of NES by a confocal microscopy experiment. As shown in Figure 3A, GFP-NES-*HopA1_Pss61_* failed to localize in the nucleus, while *GFP-HopA1_Pss61_* was clearly observed in the nucleus. In our repeated experiments, nuclear localization of GFP-NES-*HopA1_Pss61_* was barely detected in most cells, but it was found in a few cells (Supplementary Figure S7), suggesting residual localization of GFP-NES-*HopA1_Pss61_* in the nucleus. To optimize the effect of agrobacteria concentration on *HopA1_Pss61_*- and NES-*HopA1_Pss61_*-induced cell death response, the agrobacteria were grown to OD_600_, ranging from 0.025 to 0.2, and independently infiltrated into *N. benthamiana* and *N. tabacum* *Xanthi*. In comparison with GFP-*HopA1_Pss61_*, GFP-NES-*HopA1_Pss61_* showed a cell death response with a significantly reduced level at a final OD_600_ of 0.2 in *N. tabacum*, whereas OD_600_ was 0.05 in *N. benthamiana* (Supplementary Figure S8). As shown in Figure 3B, GFP-NES-*HopA1_Pss61_* showed a dramatically weakened cell death response, while GFP-*HopA1_Pss61_* induced strong cell death. Consistent with the visible phenotypes, GFP-NES-*HopA1_Pss61_* produced ion leakage intermediate between GFP-*HopA1_Pss61_* and negative control, GFP (Figure 3D). Western blot analysis indicated both *HopA1_Pss61_* proteins were expressed with similar levels (Figure 3C), demonstrating that the compromised cell death in GFP-NES-*HopA1_Pss61_* is not due to low levels of protein expression but due to the export of *HopA1_Pss61_* from the nucleus. Together, these results demonstrate that the nuclear pool of *HopA1_Pss61_* is required for RPS6 recognition and cell death induction.

### 2.4. RPS6 Localizes to Nucleus and Cytoplasm

**Figure 4:**
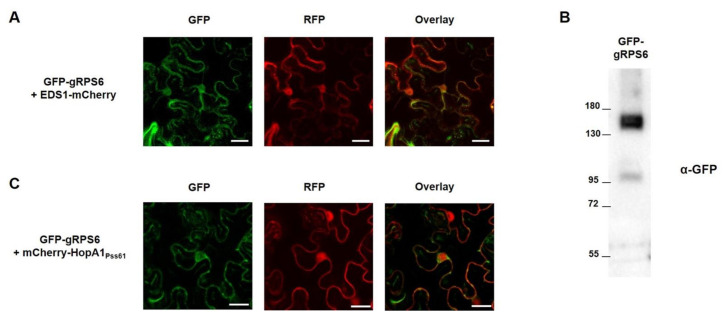
RPS6 localizes to the nucleus and cytoplasm. GFP-gRPS6 was transiently expressed with (**A**) EDS1-mCherry or (**C**) mCherry-*HopA1_Pss61_* in *N. benthamiana* leaves using Agrobacterium adjusted to an OD_600_ of 0.5 for GFP-gRPS6 and 0.3 for EDS1-mCherry or mCherry-*HopA1_Pss61_*. Two days after infiltration, cells expressing fusion proteins were analyzed by an Olympus fluoview FV1000 confocal microscope under GFP fluorescence (left), RFP fluorescence (middle), and GFP/RFP overlay (right). Scale bar: 20 μm. (**B**) Expression of GFP-gRPS6 in samples shown in (**A**) was confirmed by Western blot with anti-GFP antibody.

To elucidate the subcellular localization of RPS6 inside the plant cell, genomic RPS6 from Col-0 was fused in frame with green fluorescent protein (GFP) at the N-terminus under control of the strong CaMV 35S promoter to generate GFP-gRPS6. GFP-gRPS6 and control EDS1-mCherry were transiently expressed in *N. benthamiana* leaf cells, and their localization was monitored under the confocal microscope. As shown in Figure 4A, GFP-gRPS6 represented nucleocytoplasmic distribution and co-localized with EDS1-mCherry, which is known to localize in nucleus and cytoplasm [36], reminiscent of the pattern of HopA1 localization. The expression of GFP-gRPS6 was confirmed by Western blot analysis (Figure 4B), suggesting the localization of RPS6 was based on full-length protein expression. Since *HopA1_Pss61_*-triggered cell death requires nuclear localization of *HopA1_Pss61_*, we hypothesized that this localization is important for RPS6 activation. To test the hypothesis, we co-expressed GFP-gRPS6 and mCherry-*HopA1_Pss61_* in *N. benthamiana* leaf cells. As expected, we observed the co-localization of GFP-gRPS6 and mCherry-*HopA1_Pss61_* in the nucleus and cytoplasm (Figure 4C). RPS6 recognizes *HopA1_Pss61_* indirectly, as *HopA1_Pss61_* did not interact with RPS6 in yeast-two hybrid analysis (Supplementary Figure S9). Together with Figure 3, these results suggest that the indirect recognition of *HopA1_Pss61_* by RPS6 in the nucleus might be necessary to activate ETI responses.

### 2.5. Induction of HopA1_Pss61_ Triggers Defense Responses and Bacterial Growth Suppression in Arabidopsis

**Figure 5:**
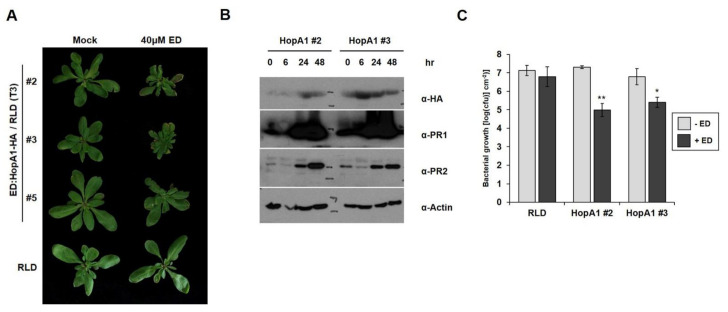
Induction of *HopA1_Pss61_* triggers defense responses and bacterial growth suppression. (**A**) Estradiol-*HopA1_Pss61_* transgenic plants were generated in wild-type RLD. Plant phenotype was analyzed 7 days after spraying 40 μM estradiol (ED) or ethanol (Mock); (**B**) Total proteins were isolated in estradiol-*HopA1_Pss61_* transgenic plants (line #2 and line #3) at the indicated times after spraying estradiol. Immunoblots were analyzed with the indicated antibodies; (**C**) In planta bacterial growth was measured in indicated plants after inoculation with DC3000 at 10^5^ cfu/cm^2^ in the absence (gray columns) or presence (black columns) of estradiol. Asterisk indicates significant differences with wild-type RLD (** *p* < 0.01, * *p* < 0.05, Student’s *t*-test). This experiment was repeated twice with a similar result.

*HopA1_Pss61_* induced bacterial disease resistance and an HR response in wild-type RLD [25,26]. To gain further insight into *HopA1_Pss61_*-induced defense responses, we produced transgenic RLD lines that expressed *hopA1* under the control of an estradiol-inducible promoter, which enabled us to activate RPS6 conditionally, and selected three independent estradiol-*hopA1* homozygous lines of T3 generation (line #2, line #3, and line #5). In the absence of estradiol induction, no morphological differences were found between RLD and line #5, while line #2 and line #3 were slightly smaller than RLD. However, growth reduction and cell death-like symptoms appeared strongly in line #2 and line #3 but weakly in line #5 7 days after spraying 40 μM estradiol (Figure 5A). Consistent with the morphological phenotypes, accumulation of *HopA1_Pss61_* was detected in line #2 and line #3, whereas it was below the detection limit in line #5 (Supplementary Figure S10). In addition, levels of the defense marker proteins PR1 and PR2 gradually increased and reached a higher level at 48 h after estradiol treatment in *hopA1* transgenic lines (Figure 5B). Both induction of cell death and accumulation of PR proteins correlated with the expression level of *HopA1_Pss61_*. The basal level of cell death symptoms and PR protein expression in the mock-treated plants is likely due to a leaky expression of *HopA1_Pss61_* from estradiol-inducible promoters (Figure 5A and Figure S10) [37]. Bacterial growth assays showed that the growth of DC3000 in lines #2 and #3 was less than in RLD by a factor of 50–100 (Figure 5C). Collectively, these data indicate that defense responses are being activated in *hopA1* transgenic lines.

## 3. Discussion

Previously, we identified *RPS6* encoding a TIR-NBS-LRR (TNL) protein by using a loss of resistance screen and a positional cloning approach. RPS6 specifically recognizes a bacterial effector gene *hopA1_Pss61_* from *P. syringae* pv. *syringae* strain 61 to trigger an immune response [25,26]. *RPS6* enables genetic tools to test the range of the effector-spectrum in *srfr1*-mediated resistance in the RLD background. Mutations in *SRFR1* increase both AvrRps4- and HopA1-triggered immunity [26,38]. In addition, SRFR1 physically interacts with some TNL proteins, such as RPS4, RPS6, SNC1, and a central immune regulator, EDS1 [39,40], demonstrating a possible role of SRFR1 as a general negative regulator in TNL R protein-mediated immunity. To date, *RPS4* and *RPS6* are the only Arabidopsis TIR-NBS-LRR *R* genes for which *Pseudomonas* effector genes are known, both of which are EDS1- and SRFR1-dependent. Therefore, RPS6 allows a direct comparison with RPS4 to dissect the EDS1-dependent signaling pathway in Arabidopsis.

### 3.1. Investigation of Genes in RPS6 Locus

Some R proteins have formed paired immune receptors for the effector recognition. For example, Arabidopsis TNL R protein RPS4 genetically and physically interacts with RRS1, another TNL R protein with a WRKY domain, and is involved in the recognition of PopP2 from *Ralstonia solanacearum* and AvrRps4 from *P. syringae* [41,42,43]. RGA4-RGA5 is another example of paired receptors to detect both *Magnaporthe oryzae* effector AVR1-CO39 and unrelated *M. oryzae* effector AVR-pia [44,45]. This evidence led us to suspect whether RPS6 requires another R protein to fully recognize *HopA1_Pss61_*. Hence, we first focused on genes around *RPS6*, including six *TNL* genes and *At5g46460*, which encodes a pentatricopeptide repeat protein (Figure 1A). *At5g46460* and *RPS6 (At5g46470)* possess a short intergenic region and transcribe polycistronically as a single transcript [27]. Additionally, both *At5g46460* and *RPS6* were included in the construct for the previous RLD accession *rps6-1* complementation [26]. In Figure 1D, however, none of Col-0 T-DNA insertion lines, except *rps6-3* and *rps6-4*, abolish *HopA1_Pss61_*-triggered immunity, indicating that RPS6 indeed recognizes *HopA1_Pss61_* in Col-0 as well as RLD, and does not require other surrounding genes for *HopA1_Pss61_* recognition. The current gene model in TAIR10 showed that *RPS6* contains an extensive (~3 kb) 3′ UTR with six exons. The long 3′ UTR is a typical characteristic of nonsense-mediated mRNA decay (NMD) target [28]. *RPS6* is required for autoimmunity in NMD-deficient mutant *smg7* [29]. Moreover, aberrant transcripts were expressed in the 3′ UTR region of *RPS6* in the absence of *SMN2*, which encodes DEAD-Box RNA Helicase [46]. In our pathogenesis assay, *rps6-5*, in which the T-DNA was inserted at position 1789 of 3′ UTR within exon 10, did not compromise RPS6 function (Figure 1). Consistent with this, in our previous study, we found the presence of poly-A tails within the exon 9 [26]. Together, these results raise the possibility that 1789 bp of *RPS6* 3′ UTR region is sufficient to confer *HopA1_Pss61_*-triggered resistance and might be a target of NMD that possibly controls aberrant *RPS6* transcripts to fine-tune plant growth and defense.

### 3.2. Localization and Functional Analysis of HopA1_Pss61_ and RPS6

To gain a better understanding of HopA1 function, we analyzed the subcellular localization and cell death induction of *HopA1_Pss61_* and HopA1_DC3000_. GFP-*HopA1_Pss61_* (GFP fused to the N-terminus of *HopA1_Pss61_*) was localized to the nucleus, cytoplasm, PM, and PD, and induced cell death. Surprisingly, *HopA1_Pss61_*-GFP (GFP fused to the C-terminus of *HopA1_Pss61_*) was not found in the nucleus and failed to trigger cell death for unknown reasons (Figure 2A,C). The biochemical function of *HopA1_Pss61_* is unknown, and we cannot exclude that the C-terminally tagged GFP may cause improper protein folding or occlude a nuclear localization signal (NLS) of *HopA1_Pss61_* [47]. Additionally, GFP-NES-*HopA1_Pss61_* was excluded from the nucleus, and its mislocalization leads to the loss of *HopA1_Pss61_*-induced cell death (Figure 3 and Figure S8). HopA1_DC3000_ did not produce cell death regardless of the position of the GFP tag (Figure 2A,C). Together, these results imply that the nuclear localization of *HopA1_Pss61_* is necessary to induce cell death. 

RPS6 was also found in the nucleus and cytoplasm (Figure 4). In our yeast-two hybrid assay, no physical interaction was observed between RPS6 and *HopA1_Pss61_* (Supplementary Figure S9). Again, it is likely that a nuclear pool of RPS6 and *HopA1_Pss61_* may play an essential role in ETI, as was proposed for N and RPS4 [48,49]. In addition, we cannot exclude the possibility that RPS6 and *HopA1_Pss61_* might indirectly interact in the nucleus with the help of guardee (or decoy). The nucleolus is not only involved in the biogenesis of ribosomal RNA but is also implicated in the control of disease, regulation of cell cycle, and as a storage site [50]. Fuhrman and coworkers showed that the NOL-6 nucleolar protein in *Caenorhabditis elegans* suppressed innate immunity against bacterial pathogens by inhibiting the transcriptional activity of the tumor suppressor p53 [51]. Both GFP-*HopA1_Pss61_* and GFP-HopA1_DC3000_ localized to the nucleolus (Figure 2A and Figure S3A), suggesting that both HopA1 proteins may interact with host virulence target(s) in the nucleolus to enhance bacterial virulence, whereas only *HopA1_Pss61_* is monitored by RPS6 to trigger ETI responses.

In Supplementary Figures S2 and S3, we found *HopA1_Pss61_* localized in both PM and PD. PD are known as intracellular channels in plants that offer an effective cell-to-cell exchange of signal molecules [31]. PD are in charge of chloroplast metabolism, the ER to Golgi secretion system, as well as callose-deposition, a well-established defense response involved in plant innate immunity [52,53,54]. Indeed, some effectors are reported to target PD to suppress PTI responses. For example, the *Fusarium graminearum* effector FGL1 is known to inhibit callose-mediated immunity by releasing free fatty acids [55]. *Xanthomonas campestris pv. vesicatoria* effector XopJ also suppresses callose deposition [56]. Therefore, we could not exclude the possibility that HopA1 targets PD for its virulence function. As shown in the example of AvrRps4, which promotes bacterial virulence and suppresses PTI [57], *HopA1_Pss61_* may compromise PTI in the absence of RPS6, albeit the virulence function of *HopA1_Pss61_* is unknown.

The remaining open challenge is to identify the guardee (or decoy) and/or virulence target protein(s) of *HopA1_Pss61_*. Although *HopA1_Pss61_* interacts with EDS1, it has not been shown that this is what activates RPS6 [39]. This would allow us to elucidate the molecular mechanism of *HopA1_Pss61_*-triggered immunity or its virulence function. Functional characterization of RPS6 and comparisons with RPS4 will contribute to a closer dissection of the TNL resistance pathway, which is regulated by the positive regulator EDS1 and negative regulator SRFR1.

## 4. Materials and Methods

### 4.1. Plasmid Construction

For epitope-tagged RPS6 constructs, genomic *RPS6* DNA was amplified by PCR from Col-0. BP clonase recombination reactions were carried out to insert the PCR products into the pDONR201 entry vector according to the manufacturer’s instructions (Invitrogen, Carlsbad, USA). LR reactions were performed to recombine the entry clones into the pSITE-GFP GATEWAY compatible destination vector to construct GFP-gRPS6 under the control of the CaMV 35S promoter. For epitope-tagged HopA1 constructs, the coding regions of HopA1 from *HopA1_Pss61_* and HopA1_DC3000_ were inserted into the pDONR207 entry vector. Using GATEWAY LR reactions (Invitrogen, Carlsbad, USA), we produced pMDC43-*HopA1_Pss61_* (GFP-*HopA1_Pss61_*), pMDC83-*HopA1_Pss61_* (*HopA1_Pss61_*-GFP), pMDC43-HopA1_DC3000_ (GFP-HopA1_DC3000_), pMDC83-HopA1_DC3000_ (HopA1_DC3000_-GFP), HApBA-*HopA1_Pss61_* (HA-*HopA1_Pss61_*), and pLN604-*HopA1_Pss61_* (Estradiol-*HopA1_Pss61_*-HA). For a generation of NES-*HopA1_Pss61_*, MLPPLGALTL amino acid sequence was attached in front of *HopA1_Pss61_* by PCR, as described previously [58]. mCherry-*HopA1_Pss61_*, PBS1-mCherry, and EDS1-mCherry were generated using a modified multisite Gateway cloning system (Invitrogen, Carlsbad, USA) as described [30]. PDLP5-RFP is a kind gift from Jae-Yean Kim (Gyeongsang National University).

### 4.2. Arabidopsis

Arabidopsis T-DNA insertion plants, *At5g46460* (SALK_033891), *rps6-3* (SALK_029541), *rps6-4* (SALK_204713), *rps6-5* (SALK_205232), *At5g46490* (SALK_208295), *At5g46500* (SALK_147652), *At5g46510* (SALK_205636), and *At5g46520* (SALK_084068), were obtained from Arabidopsis Biological Resource Center (ABRC). Arabidopsis wild-types, Col-0 and RLD, have been described previously [59]. *pLN604-*HopA1_Pss61_** construct was transformed into Col-0 for generating estradiol-inducible *HopA1_Pss61_* transgenic plants. 

### 4.3. Disease and Bacterial Growth Curve Assay

*Pseudomonas syringae* pv *tomato* strain DC3000 expressing the empty vector pML123 or expressing *shcA-hopA1* from *P. syringae* pv *syringae* strain 61 were described in previous studies [25,26]. For both disease and bacterial growth curve assay, Arabidopsis plants were grown under 11 h light/13 h dark cycle at 70% humidity and 21 °C conditions. For disease assays, 4-week-old Arabidopsis leaves were infiltrated with a bacterial suspension of 5 × 10^6^ colony-forming units cfu/mL in 10 mM MgCl_2_ using a 1 mL needless syringe. For in planta bacterial growth assays, bacterial suspensions of 2 × 10^5^ cfu/mL were infiltrated into leaves of 4-week-old plants. After 3 days, two leaf discs (a total of 0.5 cm^2^) were collected by cork borer (model: KA-48, size: 5) and ground in 10 mM MgCl_2_ and plated in serial dilution on Pseudomonas Agar F (MB cell, Seoul, Korea) with appropriate antibiotics, all in quadruplicate at the indicated time points.

### 4.4. HR and Ion Leakage Assays

For HR assay, *N. benthamiana* or *N. tabacum* cv. *Xanthi* plant was grown under 9 h light/15 h dark cycle at 60% humidity and 24–26 °C condition for 5–6 weeks. HopA1 constructs were mobilized into the *Agrobacterium tumefaciens* strain C58C1 containing the virulence plasmid pCH32. After overnight culture in LB media, agrobacteria cells were pelleted and resuspended in 10 mM MgCl_2_ with 100 µM acetosyringone (Sigma-Aldrich, St. Louis, MO, USA) adjusted to an OD600 of 0.2. The Agrobacterium was incubated for 2 h at room temperature and infiltrated into *Nicotiana* species leaves with a 1 mL needleless syringe. Silencing suppresser P19 was co-infiltrated for the Agrobacterium-mediated transient expression. Cell death phenotypes were visualized 4 to 5 days post-infiltration in *N. benthamiana* and 2 days post-inoculation in *N. tabacum* cv. *Xanthi*.

Ion leakage assay was performed as described [60]. Briefly, the Agrobacterium carrying relevant constructs were infiltrated into *N. benthamiana* leaves as described above. Six leaf discs were collected at 24 h after inoculation and washed three times for 10 min with distilled water. The leaf discs were immersed in a 12-well plate containing 4 mL of distilled water. The conductivity was measured by using Traceable (R) Conductivity/TDS Meter (VWR). The time-point for ion leakage was followed as described in Figure 3D.

### 4.5. Confocal Laser Scanning Microscopy

A confocal microscopy assay was performed to monitor the subcellular localization of HopA1 and RPS6. The Agrobacterium suspension was infiltrated into 4–5-week-old *N. benthamiana* plants by routine procedures. Two days later, plant tissues for live imaging were observed with an Olympus fluoview FV1000 or Olympus FV1000MPE. The GFP and RFP fluorescence was excited by a 488 nm laser and a 559 nm argon laser, respectively. For 4′,6-diamidino-2-phenylindole (DAPI) staining, *N. benthamiana* tissues were cut into small pieces and stained in DAPI solution (Sigma-Aldrich, St. Louis, MO, USA) at a concentration of 1 µg/mL for 30 min under dark condition. DAPI signals representing for nucleus were excited by a 405 nm laser.

### 4.6. Western Blot Analysis and Protein Fractionation

For normal Western blotting, Arabidopsis and *N. benthamiana* tissues were ground in 100 µL of 8 M urea buffer to extract total protein, as described previously [40]. Plant debris was pelleted at 12,000 rpm for 10 min, and the collected supernatant was used for immunoblotting. After adding 5× loading dye to samples, the mixtures were boiled for 5 min. Protein samples were separated on an 8–10% SDS-polyacrylamide gel and were transferred onto immune-blot PVDF membrane by Trans-blot^®^ Turbo (Bio-Rad, Hercules, Wilmington, DE, USA). Immunodetection was performed with anti-HA-HRP (Roche, Basel, Switzerland), anti-GFP (Abcam, Cambridge, MA, USA), anti-PR1 (Agrisera, Vännäs, Sweden), anti-PR2 (Agrisera, Vännäs, Sweden), anti-Actin (Agrisera, Vännäs, Sweden), and anti-Rabbit-HRP (Promega, Madison, WI, USA) antibodies. Detected proteins were visualized with an ECL Plus chemiluminescent kit (Bio-Rad, Hercules, CA, USA).

Protein fractionation was performed on *N. benthamiana* expressing GFP-*HopA1_Pss61_* based on Plant Nuclei Isolation/Extraction Kit (Sigma, St. Louis, MO, USA). In detail, tissue was processed in NIB buffer. Extracts were filtered and centrifuged at 3000× *g* for 10 min at 4 °C to produce a nuclear pellet. The supernatant containing cytoplasmic proteins was transferred to a new tube and remained on ice. The nuclear pellet was resuspended in NIBA buffer and 1.5 M sucrose, then was centrifuged at 13,000× *g* for 10 min at 4 °C. The white pellet containing nuclear proteins was collected and resuspended in protein extraction buffer. Further steps are similar to that in the mentioned normal Western blotting. Immunodetection was performed with anti-GFP to detect GFP-*HopA1_Pss61_* and with anti-Histone H3 and anti-PEPC to confirm correct nuclear and non-nuclear fractions, respectively. 

### 4.7. Yeast Two-Hybrid

For yeast two-hybrid, *hopA1_Pss61_* and *RPS6* (CDS) were cloned in *pDEST32* and *pDEST22*, respectively. The *pDEST32-hopA1_Pss61_* and *pDEST22-RPS6* constructs were transformed together into yeast strain PJ69-4A by a standard yeast transformation procedure. The transformation mixture was plated on SD media (-Trp-Leu, -Trip-Leu-His). Plates were grown at 30 °C and examined 4 days later.

## 5. Conclusions

Effector-triggered immunity (ETI) is mediated by genetic interactions between plant resistance (*R*) genes and pathogen avirulence (*avr*) genes and is highly effective in protecting plants from pathogens. Although over the past 20 years, many *R* genes have been identified, the mechanism of how *R* proteins induce resistance upon a perception of cognate effector proteins is still unclear.

Here, we investigated a bacterial effector, *HopA1_Pss61_*-triggered plant immune responses. Preferentially, we showed that RPS6 is the only R protein that recognizes *HopA1_Pss61_* in Col-0. RPS6 and *HopA1_Pss61_* co-localize in the nucleus and cytoplasm. Moreover, by exporting *HopA1_Pss61_* from the nucleus with nuclear export signal (NES), we uncovered that a nuclear pool of *HopA1_Pss61_* is critical for ETI responses. Additionally, we demonstrated that transgenic Arabidopsis plants expressing *hopA1_Pss61_* with an estradiol inducible system showed a dwarf morphology, a cell death response, bacterial growth inhibition, and increased accumulation of defense marker proteins. Together, these findings suggest that plants build up an RPS6 recognition system for *HopA1_Pss61_* and that the nuclear localization of *HopA1_Pss61_* and RPS6 is involved in ETI.

Our current research to increase the understanding of plant innate immunity in the reference plant Arabidopsis can be applied to crop plants for durable pathogen resistance, which can reduce our reliance on chemical disease control and improve agricultural safety and crop yields.

## Data Availability

Not applicable.

## Supplementary Materials

The following are available online at https://www.mdpi.com/article/10.3390/plants10050888/s1. Figure S1: (**A**) Genotyping analysis from 3 independent T-DNA lines of *RPS6*. Amplicons were obtained from LP, RP, and LB primers combination. (**B**) RT-PCR analysis of *RPS6* transcripts in Col-0, *rps6-3, rps6-4* and *rps6-5*, Figure S2: Both *HopA1_Pss61_* and HopA1_DC3000_ were localized to the plasma membrane, Figure S3: *HopA1_Pss61_* was localized to the plasma membrane and plasmodesmata, Figure S4: HA-*HopA1_Pss61_* induced cell death in *N.benthamiana*, Figure S5: Expression of GFP-*HopA1_Pss61_*, *HopA1_Pss61_*-GFP, GFP-HopA1_DC3000_ and HopA1_DC3000_-GFP in samples shown in Figure 2A,B was confirmed by western blot with anti-GFP antibody, Figure S6: HopA1Pss61 was accumulated in the nucleus and cytoplasm, Figure S7: Residual localization of GFP-NES-HopA1Pss61 in the nucleus, Figure S8, GFP-NES-HopA1pss61 partially suppresses cell death, Figure S9: *HopA1_Pss61_* does not interact with RPS6 in yeast two-hybrid assay, Figure S10: Induction of *HopA1_Pss61_* triggers accumulation of defense-related proteins, Table S1: Primers used in this study.
