# Supplementary material for: Nuclear Localization of HopA1Pss61 Is Required for Effector-Triggered Immunity
Source: Plants (Basel). 2021 Apr 28;10(5):888. doi: 10.3390/plants10050888 (PMC8145104; doi:10.3390/plants10050888)
Supplement: Supplementary file 1 [file plants-10-00888-s001.zip › plants-1168922-supplementary.pdf]

plants

Supplementary materials

Nuclear Localization of HopA1Pss61 Is Required for Effector-Triggered Immunity

Hobin Kang [1], Quang-Minh Nguyen [1], Arya Bagus Boedi Iswanto [1], Jong Chan Hong [1,2], Saikat Bhattacharjee [3], Walter Gassmann [4] and Sang Hee Kim [1,2,*]

[1] Division of Applied Life Science (BK21 Four Program), Plant Molecular Biology and Biotechnology Research Center, Gyeongsang National University, 501 Jinju-daero, Jinju 52828, Korea; he-good123@naver.com (H.K.); nguyenquangminh4595@gmail.com (Q.-M.N.); aryabagus62@gmail.com (A.B.B.I.); jchong@gnu.ac.kr (J.C.H.)
[2] Division of Life Science, Gyeongsang National University, 501 Jinju-daero, Jinju 52828, Korea
[3] Laboratory of Signal Transduction and Plant Resistance, UNESCO—Regional Centre for Biotechnology (RCB), NCR—Biotech Science Cluster, 3rd Milestone, Faridabad-Gurgaon Expressway, Faridabad-121 001, India; saikat@rcb.res.in
[4] Division of Plant Sciences, Christopher S. Bond Life Sciences Center and Interdisciplinary Plant Group, University of Missouri, Columbia, MO 65211, USA; gassmannw@missouri.edu
* Correspondence: sangheekim@gnu.ac.kr

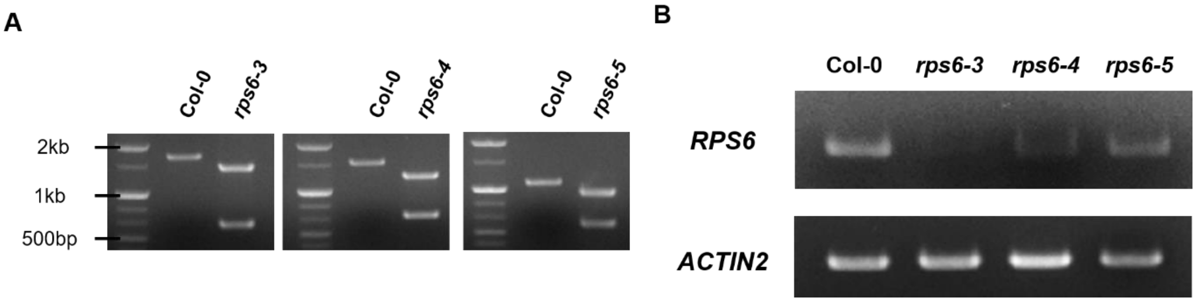

Supplementary Figure S1. (**A**) Genotyping analysis from 3 independent T-DNA lines of *RPS6*. Amplicons were obtained from LP, RP, and LB primers combination. (**B**) RT-PCR analysis of *RPS6* transcripts in Col-0, *rps6-3*, *rps6-4* and *rps6-5*. *ACTIN2* was used as a loading control. *RPS6*-specific primers used to amplify transcripts are listed in Table S1.

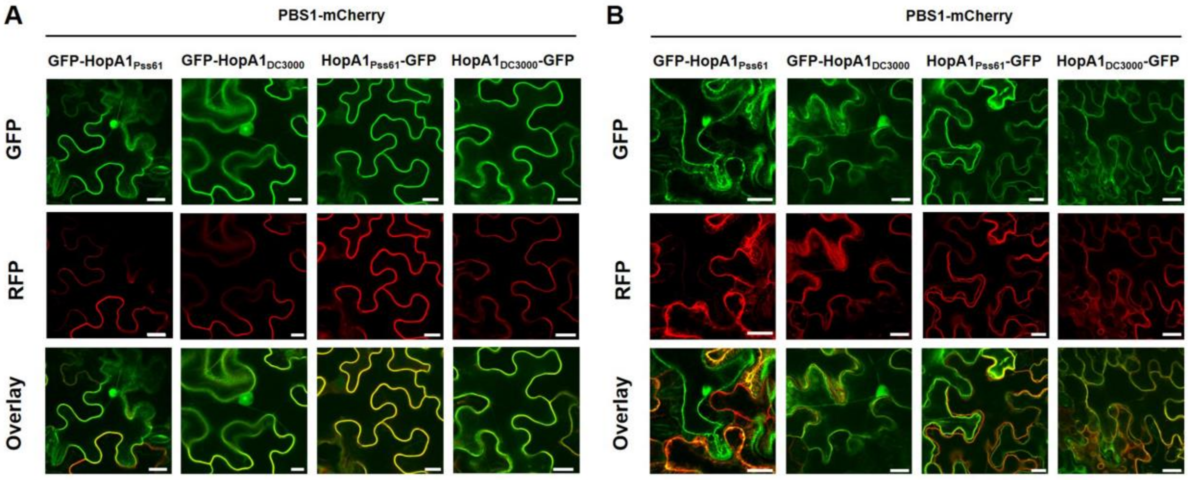

Supplementary Figure S2. Both HopA1$_{Pss61}$ and HopA1$_{DC3000}$ were localized to the plasma membrane. *GFP-hopA1*$_{Pss61}$, *hopA1*$_{Pss61}$*-GFP*, *GFP-hopA1*$_{DC3000}$ and *hopA1*$_{DC3000}$*-GFP* fusion constructs were transiently expressed in *N. benthamiana* leaves with *PBS1-mCherry,* a PM marker gene. Localization of both HopA1 was verified in the absence (**A**) or the presence (**B**) of 0.8 M Mannitol treatment. Yellow fluorescence indicates a co-localization between HopA1s and PBS1. Scale bar: 20 μm.

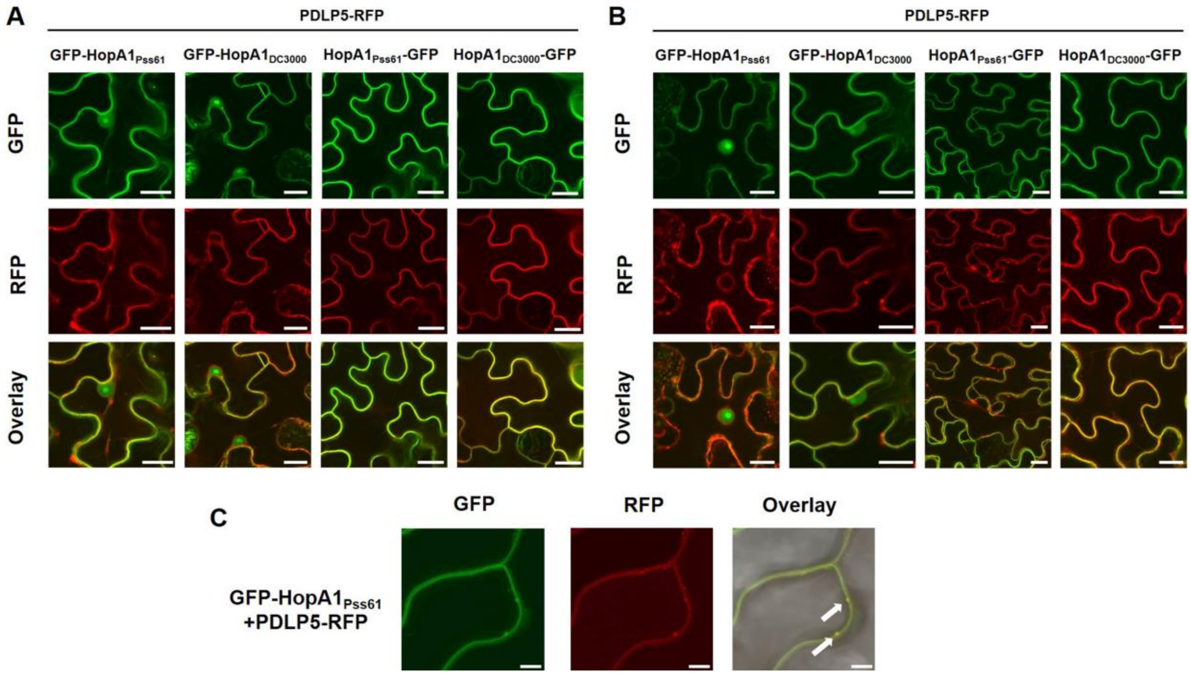

Supplementary Figure S3. HopA1$_{Pss61}$ was localized to the plasma membrane and plasmodesmata. *GFP-hopA1*$_{Pss61}$, *hopA1*$_{Pss61}$*-GFP*, *GFP-hopA1*$_{DC3000}$ and *hopA1*$_{DC3000}$*-GFP* fusion constructs were transiently expressed in *N. benthamiana* leaves with *RFP-PDLP5,* a PM and PD marker gene. Localization of both HopA1 was verified in the absence (**A**) or the presence (**B**) of 0.8 M Mannitol treatment. Yellow fluorescence indicates a co-localization between HopA1s and PDLP5. Scale bar: 20 μm.

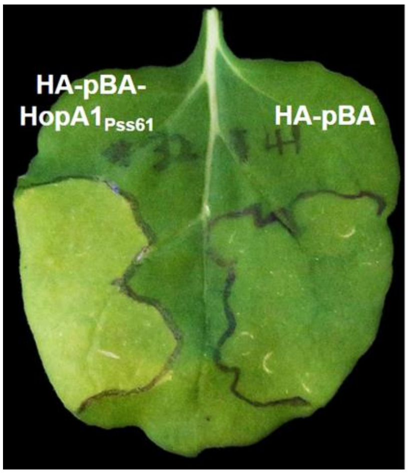

Supplementary Figure S4. HA-HopA1$_{Pss61}$ induced cell death in *N.benthamiana*. HA-pBA-HopA1$_{Pss61}$ and HA-pBA were transiently expressed in *N.benthamiana*. Agrobacterium adjusted to an OD_{600} of 0.2. Phenotypes were recorded at 4 days post-inoculation.

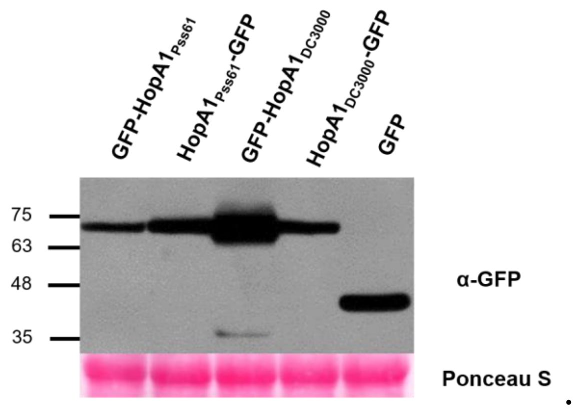

Supplementary Figure S5. Expression of GFP-HopA1$_{Pss61}$, HopA1$_{Pss61}$-GFP, GFP-HopA1$_{DC3000}$ and HopA1$_{DC3000}$-GFP in samples shown in Figures 2A and 2B was confirmed by western blot with anti-GFP antibody. Total protein was extracted from four-leaf discs at 2 days post inoculation. Total protein staining (Ponceau S) confirmed equal loading in western blot analysis.

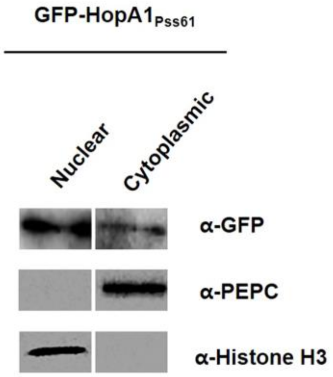

Supplementary Figure S6. HopA1$_{Pss61}$ was accumulated in the nucleus and cytoplasm. HopA1$_{Pss61}$-inoculated samples were separated into the nuclear and cytoplasmic fractions. Expression of HopA1$_{Pss61}$ in each fraction was detected by western blot. Phosphoenolpyruvate carboxylase (PEPC) and Histone H3 were used as cytoplasmic and nuclear marker proteins, respectively. The nuclear fraction is ten-fold concentrated compared with the cytoplasmic fraction.

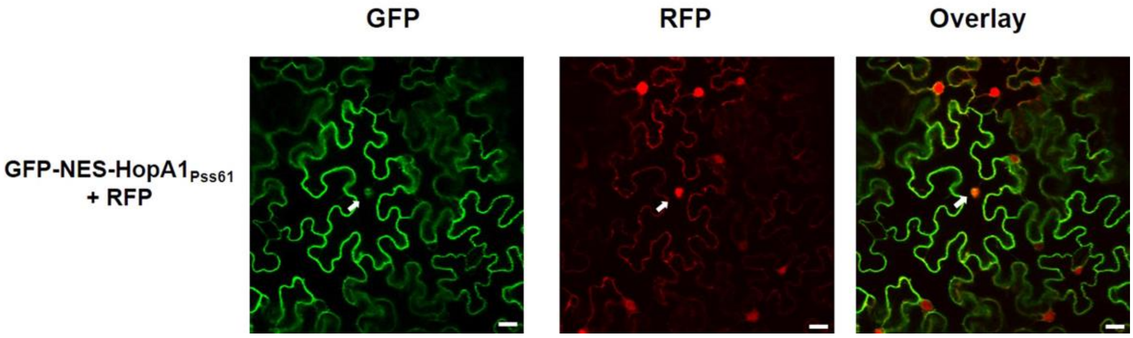

Supplementary Figure S7. Residual localization of GFP-NES-HopA1$_{Pss61}$ in the nucleus. Nuclear localization of GFP-NES-HopA1$_{Pss61}$ was found in a few cells (less than one nuclear localization among ten transformations). *GFP-hopA1*$_{Pss61}$ or *GFP-NES-hopA1*$_{pss61}$ constructs with *RFP* were transiently expressed in *N. benthamiana* leaves using Agrobacterium adjusted to an OD_{600} of 0.2. Cells expressing fusion proteins were analyzed 2 days after infiltration by an Olympus fluoview FV1000 confocal microscope under GFP fluorescence (left), RFP fluorescence (middle) and GFP/RFP overlay (right). Scale bar: 20 μm.

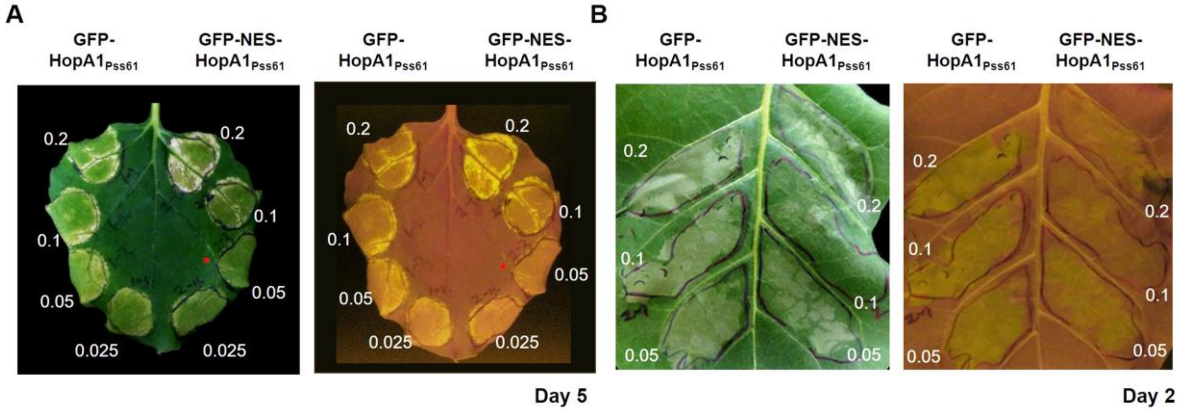

Supplementary Figure S8. GFP-NES-HopA1$_{pss61}$ partially suppresses cell death. *GFP-hopA1*$_{Pss61}$ and *GFP-NES:hopA1*$_{pss61}$ constructs were transiently expressed in (**A**) *N. benthamaina* using Agrobacterium adjusted OD_{600} ranging from 0.025 to 0.2 and (**B**) *N. tabacum* cv. Xanthi using Agrobacterium adjusted to an OD_{600} ranging from 0.05 to 0.2. The photographs were taken under visible light (left) and UV light (right) at (**A**) 5 days post inoculation and (**B**) 2 days post inoculation.

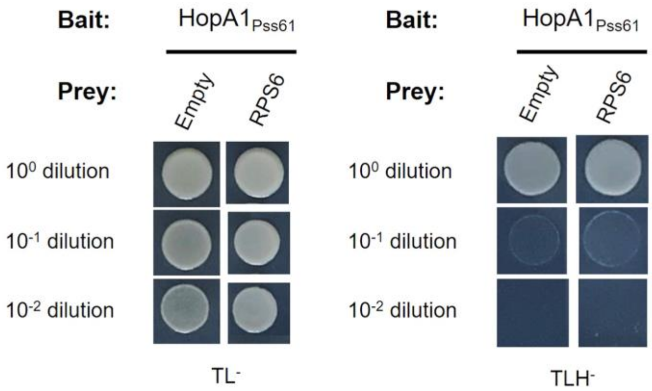

Supplementary Figure S9. HopA1$_{Pss61}$ does not interact with RPS6 in yeast two-hybrid assay. HopA1$_{Pss61}$ and RPS6 were used as bait and prey, respectively. -TL, medium lacking Trp and Leu (selection for prey and bait vectors); -TLH, medium lacking Trp, Leu and His (selection for bait and prey interaction).

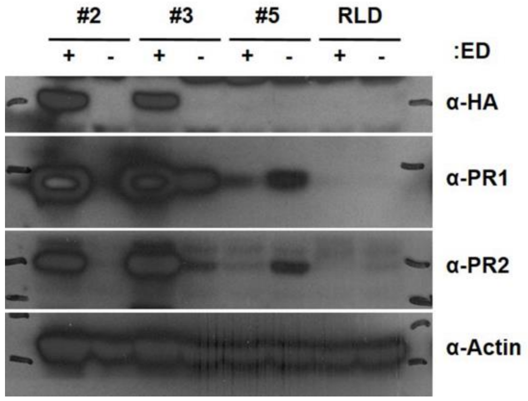

Supplementary Figure S10. Induction of HopA1$_{Pss61}$ triggers accumulation of defense-related proteins. Total proteins were isolated in estradiol-HopA1$_{Pss61}$ transgenic plants (line #2, line #3 and line #5) at 48h after spraying estradiol (+) or ethanol (-). Immunoblots were analyzed with the indicated antibodies.

Supplementary Table S1. Primers used in this study

Primer	Sequences (5′-3′)	Purpose
46460 LP	CTCTGGAACTTTGCCGTTC	Genotyping
46460 RP	CCCAACTTGATGATCAAGCC	Genotyping
rps6-3 LP	TGCCTTAACGAGCTACTGGA	Genotyping
rps6-3 RP	ATAGTCGAAGCGTTCCGGTAAGT	Genotyping
rps6-4 LP	ACTTACCGGAACGCTTCGACTAT	Genotyping
rps6-4 RP	CCCCACTGGTACGGTGAGTG	Genotyping
rps6-5 LP	GGAGAGTGGCGTTGGCTG	Genotyping
rps6-5 RP	GGACTCCCTAATTGCATGT	Genotyping
46490 LP	GCTGGTGTCTTAACGAGTTGC	Genotyping
46490 RP	CTACCCCGCAGATACTTACCC	Genotyping
46500 LP	GGCTTCAGAAACATGTGGAAG	Genotyping
46500 RP	TCTTTGTCCAATACACCCTCC	Genotyping
46510 LP	GGAGAACCGACTTGGTAATCC	Genotyping
46510 RP	CGGCAGCAAAACTACTCATTC	Genotyping
46520 LP	GGCTTGAGAAACATGTGGAAG	Genotyping
46520 RP	CTTGGAGCTCTGGAATATCCC	Genotyping
LB1.3(Salk)	ATTTTGCCGATTTCGGAAC	Genotyping
RPS6 RT Forward	CCCGAGTTTCAGCGGAGAAG	RT-PCR
RPS6 RT Reverse	AATGGTCGTTTTGCCAATTC	RT-PCR
Actin 2 Forward	TCGGTGGTTCCATTCTTGCT	RT-PCR
Actin 2 Reverse	GCTTTTTAAGCCTTTGATCTTGAGAG	RT-PCR
NES-HopA1 Forward	ATGCTGCCTCCTCTGGAAC-GCCTGACTCTGGTGAACCCTATCCATGCACG	Add NES Sequence in front of HopA1
GW-NES-HopA1 Forward	GGGGACAAGTTTGTACAAAAAA-GCAGGCTCAATGCTGCCTCCTCTGGAAC-GCCTGACTCTG	Use in Gateway system
GW-NES-HopA1 Reverse	GGGGACCACTTTGTACAAGAAAGCTGGGTATCAG-TTTCGCGCCCTGAG	Use in Gateway system
